# Looking back on the first year of *Neural Systems & Circuits*

**DOI:** 10.1186/2042-1001-2-1

**Published:** 2012-01-05

**Authors:** Peter E Latham, Venkatesh N Murthy

**Affiliations:** 1 Gatsby Computational Neuroscience Unit, UCL, London, UK; 2 Department of Molecular and Cellular Biology, and Center for Brain Science, Harvard University, Cambridge, MA, USA

## 

This month, *Neural Systems & Circuits *[1] is one year old, and we are very grateful for the support and encouragement we have received from the neuroscience community over the last year. There are many things to celebrate: we have been accepted by PubMed (so your articles will actually be indexed!), we recently published a thematic series on invertebrate circuitry [2] a sometimes underappreciated, but very important, area of neuroscience, and we have initiated an "Opinionated Neuroscientists" series [3], so those late night discussions in dingy bars can see the light of day.

In our inaugural Editorial [4], we noted our desire to foster communication among theorists and experimentalists. Promisingly, computational and experimental papers have taken residence side by side in the first year. Research articles spanned a broad range, both in technique and organism. Computational work has included analysis of electrical coupling among neurons in the cerebellum and papers on visual cortical circuits. Exciting synthesis of computation and experiment was exemplified by work that compared the actual connectivity in an invertebrate network to that predicted by a popular method that uses the so-called Granger causality - the conclusions were not very favorable to Granger causality. Experimental work included analysis of synapse formation in the adult mouse olfactory bulb and development of a specific interneuron in the Drosophila brain. The explosive development of techniques in neuroscience was illustrated by papers describing new methods and reagents.

We are hopeful that *Neural Systems & Circuits *will continue to grow in 2012, and are always keen to discuss your latest findings and plans for publication. We are also thrilled to give voice to younger scientists through the opinion pieces as well as through reports from exciting meetings.

By all indications, interest in neural circuits has never been greater and the audience is enormous. Because of its open access policy, publishing new developments in *Neural Systems & Circuits *will guarantee wide readership. We invite you to submit your work so it can be read by a diverse audience comprising theorists and experimentalists, all interested in understanding how neural circuits do their magic.

## References

[B1] Neural Systems & Circuits http://www.neuralsystemsandcircuits.com

[B2] Kemenes G Editorial to the thematic series 'Invertebrate Circuitry' Neural Systems & Circuits 2011 1 10 10.1186/2042-1001-1-10 PMC3278470 22330774

[B3] Webb A Latham P Murthy V Wanted: opinionated neuroscientists Neural Systems & Circuits 2011 1 14 10.1186/2042-1001-1-14 PMC3278392 22329890

[B4] Latham PE Murthy VN Welcome to *Neural Systems and Circuits: *bridging the gap between theory and experiment Neural Systems & Circuits 2011 1 1 10.1186/2042-1001-1-1 PMC3269226 22330757

